# Sustainable Use of Sludge from Industrial Park Wastewater Treatment Plants in Manufacturing Lightweight Aggregates

**DOI:** 10.3390/ma15051785

**Published:** 2022-02-26

**Authors:** Chao-Wei Tang, Chiu-Kuei Cheng

**Affiliations:** 1 Department of Civil Engineering and Geomatics, Cheng Shiu University, No. 840, Chengching Rd., Niaosong District, Kaohsiung 83347, Taiwan; 2 Center for Environmental Toxin and Emerging-Contaminant Research, Cheng Shiu University, No. 840, Chengching Rd., Niaosong District, Kaohsiung 83347, Taiwan; 3 Super Micro Mass Research and Technology Center, Cheng Shiu University, No. 840, Chengching Rd., Niaosong District, Kaohsiung 83347, Taiwan; 4 Department of Agribusiness Management, National Pingtung University of Science & Technology, No. 1, Shuefu Rd., Neipu 91201, Taiwan; sindy@mail.npust.edu.tw

**Keywords:** lightweight aggregate, sludge, particle density, bulk density, water absorption, crushing strength

## Abstract

The aim of this study was to investigate the development of a process for manufacturing lightweight aggregates (LWAs) by incorporating sludge from wastewater treatment plants in industrial parks with reservoir sediments. The research was divided into two stages: laboratory-scale firing and large-scale firing. In the laboratory-scale stage, a high-temperature furnace was used for trial firing. In the large-scale stage, a commercial rotary kiln was used for trial firing for mass production. The test results showed that the water absorption, dry loose bulk density, and crushing strength of the sintered LWAs were 14.2–26.9%, 634–753 kg/m^3^, and 1.29–2.90 MPa, respectively. Moreover, the water absorption of the sintered LWAs increased as the percentage of added sludge increased. In addition, the dry loose bulk density of the sintered LWAs gradually decreased as the percentage of added sludge increased. Moreover, the results of the heavy metal toxicity characteristic leaching procedure (TCLP) dissolution test for the LWAs produced by blending 30–50% sludge were all lower than the standard value required by the Taiwan Environmental Protection Agency for general industrial waste. The strength grade of the sintered LWAs was 20 MPa. From this point of view, the sintered LWAs that were studied under the test conditions could be used as aggregates for lightweight concrete and would allow it to have a reasonable strength of greater than 20 MPa.

## 1. Introduction

In an effort to develop the national economy, the total number of industrial parks established in Taiwan reached 70 by 2019. The Industrial Bureau of the Ministry of Economic Affairs of Taiwan set up 39 wastewater treatment plants to ensure that the wastewater that is discharged from factories in these industrial parks complies with Taiwan’s water pollution prevention and control regulations. By removing pollutants from the wastewater, these wastewater treatment facilities assist manufacturers in these industrial parks in the treatment of the wastewater that is generated by their processes. However, after solving the problem of wastewater discharge in these industrial parks, a large amount of industrial wastewater sludge is produced, with an output of about 600,000 tons per year, thus forming a new waste treatment problem [1]. In particular, due to a variety of problems, such as difficult land acquisition and energy consumption, the disposal of waste sludge in industrial parks via landfills or incineration is not in line with current environmental goals, and it is not conducive to the construction of a recycling society. In fact, wastewater sludge contains a large number of inorganic substances, such as calcium, silicon, aluminum, iron, and other oxides that are common in nature, which have the potential to be used as a material if it is properly recycled. Considering the lack of natural resources in Taiwan and the increasing difficulty in finding waste disposal sites, the material application of wastewater sludge from industrial parks is a promising alternative.

Lightweight aggregate (LWA) is a natural or synthetic material with a dry particle density of less than 2000 kg/m^3^ or a bulk density of less than 1200 kg/m^3^ [2,3]. As with ordinary aggregates, LWAs can be divided into coarse and fine aggregates. The particle size of a lightweight coarse aggregate is greater than or equal to 5 mm, and its bulk density is less than 1000 kg/m^3^; the particle size of a lightweight fine aggregate is less than 5 mm; and the upper bulk density limit varies depending on whether it is natural or artificial, as shown in Table 1 [4]. LWA has received extensive attention due to its superior physical properties [5]. In particular, LWAs can be used to replace ordinary aggregates to produce lightweight aggregate concrete (LWAC) [2]. The American Concrete Institute (ACI) divides LWAC into three grades according to unit weight and strength: low-density concrete, medium-strength concrete, and structural concrete [6]. Low-density LWAC is generally used for thermal insulation in buildings, whereas medium-strength LWAC is often used for concrete blocks and other structures that only require a small amount of strength, and structural LWAC is used for structures that require a high amount of strength. LWAC has the advantages of being light weight and having good heat insulation, strong fire resistance, good seismic resistance, and sufficient strength [7]. As a result, LWAC has been widely used in civil construction projects, including in buildings, structures, geotechnical engineering, roads, precast members, etc.

LWAs can come from natural sources or can be made artificially [8]. The basic physical properties of common LWAs are shown in Table 2 [4]. Natural LWAs from different sources have different properties, such as different particle densities, bulk densities, and water absorption capacities. Initially, artificial LWAs tended to be made of natural raw materials such as shale, clay, slate, and phyllite [8]. However, with the growing concerns about sustainability, a wide range of urban and industrial waste types have been tested for their ability to partially or completely replace conventional raw materials for LWA production. These unconventional raw materials include ground granulated blast furnace slag [9], fly ash [10], incinerated ash [11,12,13,14], waste glass [15], sewage sludge [16,17,18,19], screen glass and polishing sludge [20], reservoir sediments [21,22], waste TFT-LCD glass powder and reservoir sediments [23], tile grinding sludge with reservoir sediments [24], waste drill cuttings [25], coal fly ash and waste glass [26], stone cutting sludge, plastic wastes, and sepiolite rejections [27], and clays and alternative raw materials [28]. Generally speaking, synthetic aggregates with a bulk density of 0.88–1.12 g/cm^3^ can be used as structural concrete; aggregates with a bulk density of less than 0.88 g/cm^3^ are mostly used to make thermal insulation elements. Artificial LWAs are mainly produced by sintering materials with expansive properties due to pyroplastic deformation [8]. Sintering occurs when powders are heated to the melting point of the main components in the green body; the powders promote close adherence between particles via various diffusion methods so as to achieve solidification, densification, recrystallization, and bonding [29]. As a result, a high-strength sintered body is produced. The artificial LWA production process includes raw material extraction, raw material processing, thermal treatment, screening, and storage. Among these processes, raw material processing and thermal treatment are the two most important steps. In particular, the bloating phenomenon that is observed in LWA at high temperatures is a key point because the expansion degree reflects its microstructure and mechanical strength, which largely control the quality of the finished product [30].

During heat treatment, the chemical and mineral compositions of the raw materials play a key role, as the production of gases and the quantity and viscosity of the glassy phase are influenced by the composition of the prepared mixture [31]. The LWA expansion theory proposed by Riley in 1951 [32] is currently the most accepted theory. He proposed that the raw material, which can produce expanded LWA, must meet two requirements. One is that the material must produce a high-temperature glassy phase with a sufficiently high viscosity to trap the gases are discharged upon mineral decomposition. The other is that a substance must be present to release the gases at the temperature at which the glassy phase forms. Once the above two requirements are met, the green pellet will generate a vitreous melt with tension within the expansion temperature range and will then expand as the gas escapes. Through an extensive chemical analysis of bloating and non-bloating clays, Riley [32] defined the bloating limits of the composition in a ternary (silica–alumina–fluxing) diagram. The “bloating area” on the diagram shows the ideal clay composition that satisfies the first necessary requirement for bloating. Most researchers still consider this to be an important part of the chemical design of LWAs [18]. On the other hand, regarding the viscosity of the melt when the green pellet is expanded, Moreno-Maroto et al. [33] suggested that achieving an adequate viscosity appears to be more decisive for bloating than gas release capacity. In addition, Balapour et al. [34] proposed that to prevent LWA from being deformed due to gravity during sintering, the lower limit of the viscosity of its glassy phase should be 100 Pa·s in order to retain the spherical shape of LWA.

Currently, all of the countries in the world are committed to environmental protection, and the treatment of industrial sludge has been carried out in a resource-based manner. In the past two decades, countries around the world have successfully created LWAs from industrial waste or sludge. These new LWA construction materials that do not use natural ores as raw materials meet the definition of green building and have been used in various fields, which also proves that they can achieve promising results. It is worth noting, however, that the chemical and mineralogical characteristics of these novel raw materials are often far different from the composition of the clays and shale that are traditionally used to make expanded clay aggregates. Therefore, the use of waste may significantly alter the manufacturing process to obtain the technical properties required for LWA. These changes are mainly related to the shaping and firing process because of the different plasticity and thermal behavior of unconventional raw materials [8,35,36].

Based on the above, in order to reduce Taiwan’s environmental load and to effectively utilize resources, the Industrial Bureau of Taiwan’s Ministry of Economic Affairs actively promotes the recycling of industrial wastewater sludge. This research was a recycling pilot project that was commissioned by the Industrial Bureau of the Ministry of Economic Affairs of Taiwan. In order to develop commercial firing and mass production technology for producing lightweight aggregates from industrial sludge, a laboratory-scale trial firing must be carried out first. Therefore, a series of aggregate formulations for different uses were developed for experimental-scale firing trials. After analyzing the properties of the fired aggregates, the optimal formulation of the aggregates was screened to provide a reference for subsequent commercial firing and mass production. In short, this study started from the perspective of green and sustainable development and conducted an early evaluation of the research and development of green light-weight aggregates and mass production technology as well as the research on the recycling technology of sludge from wastewater treatment plants in industrial parks. The main objectives of this study are to achieve multiple government goals such as “industry–university technology exchange”, “industrial technology upgrade”, and “sustainable development”.

## 2. Experimental Procedure

### 2.1. Experimental Program

In this study, the research was divided into two stages: laboratory-scale firing and large-scale firing. The overall procedure involves granulation, drying, and sintering. In the laboratory-scale stage, sintering was further divided into a preheating phase and a sintering phase. A series of different conditions (i.e., soaking time in the preheating phase and sintering temperature in the sintering phase) were established. Laboratory-scale sintering experiments were then carried out to analyze the thermal expansion range and the achievable particle density range as a reference for mass production. The physical and mechanical properties (i.e., particle density, water absorption, bloating index, and crushing strength) of the synthesized aggregates were subsequently assessed. After completing research trials in the laboratory, the feasibility of large-scale production of LWAs using wastewater treatment plant sludge was evaluated in a commercially available kiln. Moreover, the engineering properties (i.e., unit weight and compressive strength) of the concrete made from the produced LWAs were tested and examined.

### 2.2. Materials

**Figure 1:**
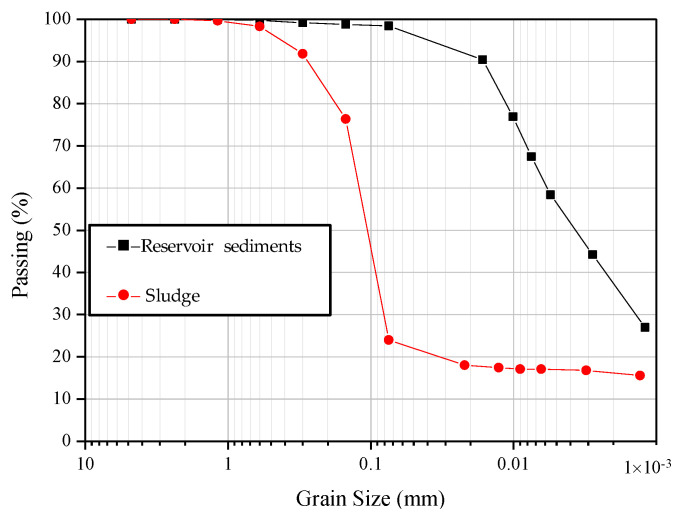
Particle size distribution curves of the materials used in the study.

Organic biological sludge from the Sinying Industrial Park Wastewater Treatment Plant, Tainan City, located in southern Taiwan was used as the raw material for the experiment. This sludge comprises general industrial waste, biological sludge, or sludge containing more than 30% volatile solids produced by biological treatment procedures. In the laboratory-scale stage, sediments from Shimen Reservoir in Taoyuan City (the third lar gest reservoir in Taiwan) were also selected for use as an additive to improve the lightweight aggregate of the industrial sludge. The physical properties and general characteristics of the wastewater plant sludge and reservoir sediments are shown in Table 3 and Table 4, respectively. Generally speaking, the plasticity of raw materials has a great influence on the production of green pellets, and its plasticity index (PI) should be greater than 7%. Among the raw materials used in this experiment, the plasticity of the sludge with PI = 4% was poor, whereas the reservoir sediments with PI = 12% had better plasticity and could meet the requirements of granulation by the plastic method. In addition, the results of the specific gravity analysis of the raw materials showed that the specific gravity of the sludge was 1.74 and that that of the reservoir sediments was 2.75, which is similar to the specific gravity of ordinary soil. The particle size distribution curve of these raw materials is shown in Figure 1. It can be seen from Figure 1 that the particle size of the sludge in the wastewater treatment plant was relatively large, with the median particle size being D_50_ ≥ 0.11 mm (indicating that more than 50% of the particles had a particle size greater than 0.11 mm). From this point of view, if batching, granulation, and firing are to yield good results, the sludge should be further refined by grinding. For the mixed reservoir sediments, particles with a particle size larger than 0.05 mm accounted for about 3%, which meets the general requirements for the particle size of raw materials.

**Figure 2:**
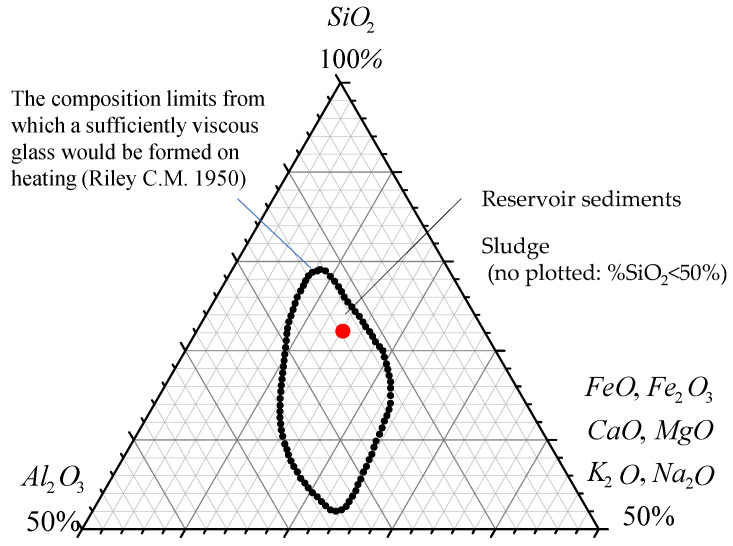
Composition limits of bloating clays according to Riley [32].

In general, the SiO_2_ and Al_2_O_3_ content will affect the formation of a highly viscous liquid phase and a trapped gas at high temperature, whereas the fluxing content (Fe_2_O_3_ + CaO + MgO + K_2_O + Na_2_O) will determine the softening and the melting temperatures of the aggregates [32]. As can be seen from Table 4, the sludge had a low SiO_2_ content and a low fluxing concentration, whereas the reservoir sediment showed a higher SiO_2_ content and a higher fluxing content. In general, if the chemical composition of the raw material used for firing LWA falls within the range of the dotted line in Figure 2, it should have a suitable melt viscosity for the expansion of the aggregate during firing. In other words, the chemical composition of the raw material is closely related to its melt viscosity. Among the raw materials used in this study, only the chemical composition of the reservoir sediments meets the requirements, whereas the industrial sludge does not fall on the dotted line graph due to its low SiO_2_ content. Therefore, it can be predicted that sludge will not provide a melt viscosity that is suitable for expansion during high-temperature firing. 

### 2.3. Preparation of Aggregate Pellets and Sintering at Laboratory Scale

According to the analysis of the physical properties of the sludge in Table 3, its plasticity is low, and without that addition of other ingredients, it is impossible to make LWA green pellets from sludge alone. Therefore, reservoir sediments were used as an admixture to assist granulation. In addition, if the rotary kiln is used for the firing process, the sludge cannot be used as the raw material for firing LWA alone because of its chemical composition. However, it can be mixed with reservoir sediments and can be modified to facilitate firing into LWA and demonstrate good performance. In other words, during high-temperature heat treatment, in order to help stabilize the salts in the sludge and to reduce the melting temperature, a large amount of modifier must be added. Therefore, in the laboratory stage, 10%, 30%, 50%, and 70% of reservoir sediments were added to the test formula for modification. According to the experimental LWA production formula proposed in Table 5, when the amount of industrial sludge reached 70%, it was impossible to produce LWA green pellets, so 50% was the upper granulation limit.

**Figure 3:**
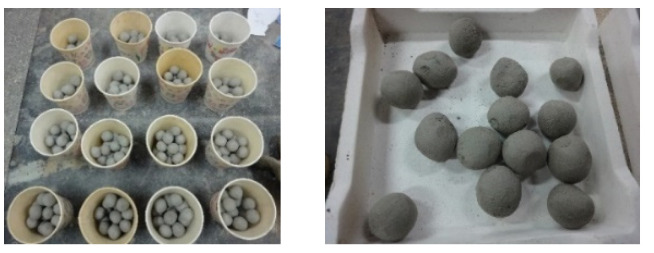
LWA green pellets.

Green pellets were produced by the plastic method; that is, homogenized raw materials with different amounts of water were added during the mixing process. The amount of mixing water was based on the amount of water with which the raw materials showed plasticity. In the laboratory stage, the mixed water-containing material was manually kneaded into aggregate pellets with diameters of about 10–12 mm, as shown in Figure 3. After that, the green pellets were dried in an oven at 105 ± 10 °C for 24 h; in addition to achieving a certain particle strength, it can also prevent explosion phenomena (cracking or steam explosion) during the firing process. After drying, the green pellets could be fired. 

**Figure 4:**
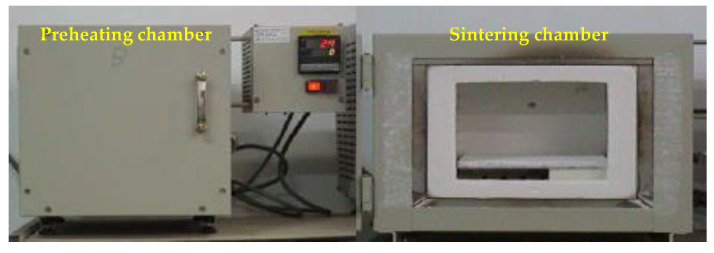
Programmable high-temperature electric furnace.

**Figure 5:**
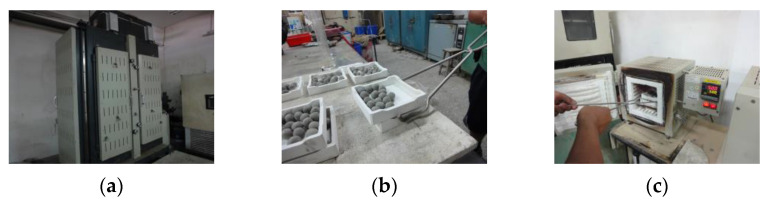

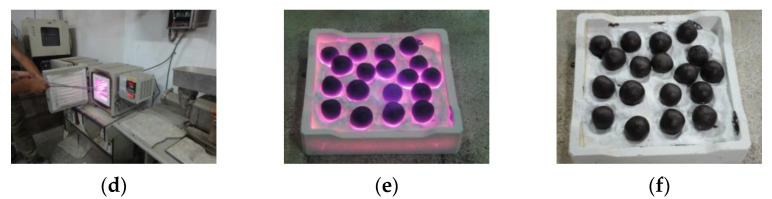
Firing process of green pellets: (**a**) put in oven to dry, (**b**) pellets after drying, (**c**) put into preheating chamber, (**d**) put into sintering chamber, (**e**) quenched in air, and (**f**) pellets after cooling.

In this study, a high-temperature heat treatment (about 1000–1250 °C) was used to fabricate the LWAs. A two-phase rapid heating procedure was adopted for the laboratory-scale firing process. This thermal pretreatment prevents the deterioration of dried pellets by rapid “flash heat”, which, in turn, prevents them from exploding and forming black cores during the final firing step [37]. The main apparatus that is used is a self-designed electric laboratory furnace, which has programmable controls and two chambers, as shown in Figure 4. During the preheating phase, the dried green pellets were placed in an aluminum oxide basin in the preheating chamber of the furnace and heated at the target temperature for different durations. It is worth noting that due to the rapid heating procedure used in this study, the preheating chamber must first be heated to the target temperature. In addition, when the sample is placed in and taken out of the preheating chamber, it must be completed in the shortest amount of time possible to avoid temperature changes from taking place in the preheating chamber. During the sintering phase, the preheated pellets were subsequently placed in the sintering chamber of the furnace, heated at the desired maximum temperatures for different durations, and then quenched in air, as shown in Figure 5. The design of the temperatures and times used for firing was set in three stages. The first-stage trial firing was to determine the sintering temperature range and softening temperature, the second-stage trial firing was to determine the soaking time in the preheating phase, and the third-stage trial firing was to further determine the physical properties of the obtained aggregates and their applicability. The steps of each stage are detailed in Section 3.1.

### 2.4. Test Methods and Data Analysis

The particle density of the sintered aggregate was determined by the Archimedes principle, as shown in the following formula [38]:

(1) $$\rho_{p}=\frac{m_{d}}{m_{s}-m_{i}}$$

where $\rho_{p}$ is the particle density (g/cm^3^), $m_{d}$ is the dry mass (g), $m_{i}$ is the immersed mass (g), and $m_{s}$ is the 24 h saturated surface-dry mass (g). 

The sintered aggregate was immersed in water for 24 h to measure its water absorption. The water absorption was calculated as follows [39]:

(2) $$\mathrm{Water}\;\mathrm{absorption}=\frac{m_{s}-m_{d}}{m_{d}}\times 100\%$$

To explore the volume change in the pellets, the bloating index was defined as the ratio of the volume of the fired pellets to the volume of the unfired green pellets, which was calculated as follows [38]:

(3) $$\mathrm{Bloating}\;\mathrm{index}=\frac{V_{a}}{V_{p}}\times 100\%$$

where $V_{p}$ (cm^3^) is the initial volume of the green pellets, and $V_{a}$ (cm^3^) is the volume of the sintered aggregate pellets. The loss on ignition was defined as the mass loss of the dried pellets after firing and was expressed as a percentage of the total initial mass. In addition, the dry loose bulk density and crushing strength of the synthetic aggregates were carried out according to CNS 1163 [40] and CNS 14779 [41], respectively. The results for each experiment in this study are the average of 10 to 15 samples.

## 3. Results and Discussion

### 3.1. Laboratory-Scale Firing

#### 3.1.1. The First-Stage Trial Firing

**Figure 6:**
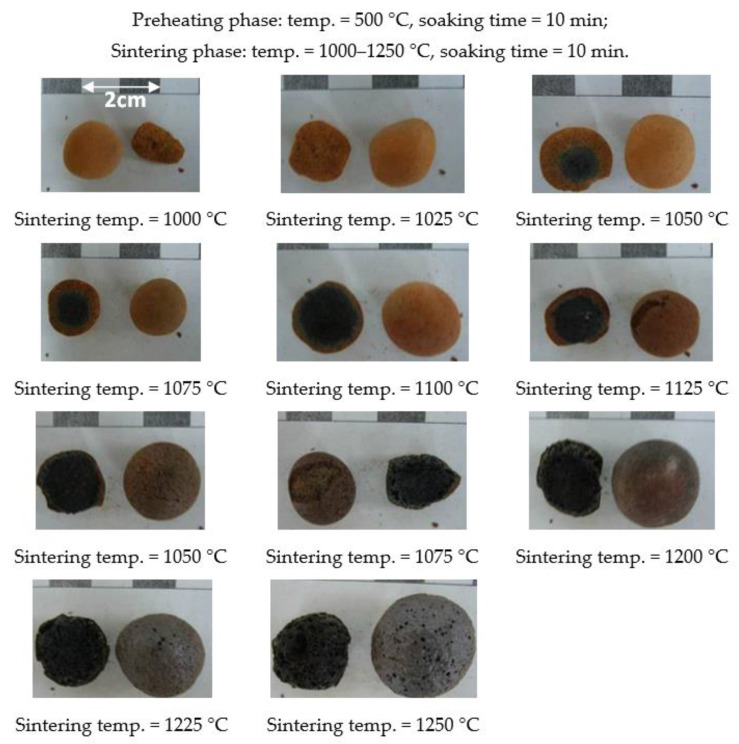
Appearances and profiles of the sintered LWAs during the first stage (the W30 formula).

**Figure 7:**
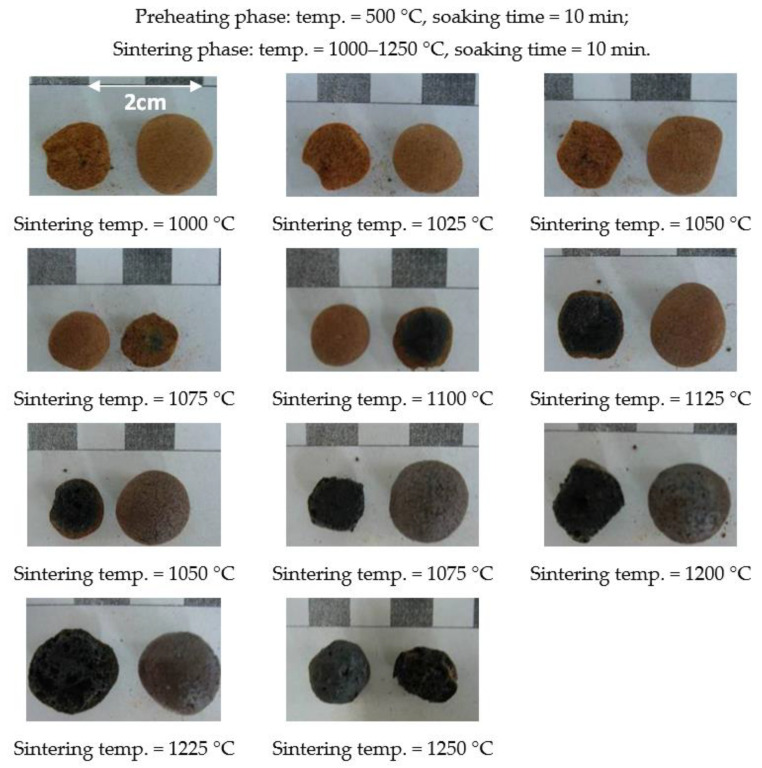
Appearances and profiles of sintered LWAs during the first stage (the W50 formula).

According to the aforementioned formula design, granulation is not possible when the amount of industrial sludge exceeds 50%. Therefore, the experiments carried out with the addition of 30% or 50% at this stage were. The trial firing conditions at this stage were a fixed preheating temperature of 500 °C and a fixed soaking time of 10 min. Under the aforementioned conditions, the sintering temperature varied between 1000 and 1250 °C, and the soaking time was set to 10 min. The determination of the sintering temperature range and the softening temperature was mainly based on the morphological changes before and after green pellet firing. Figure 6 and Figure 7 show the results of the first-stage trial firing.

**Figure 8:**
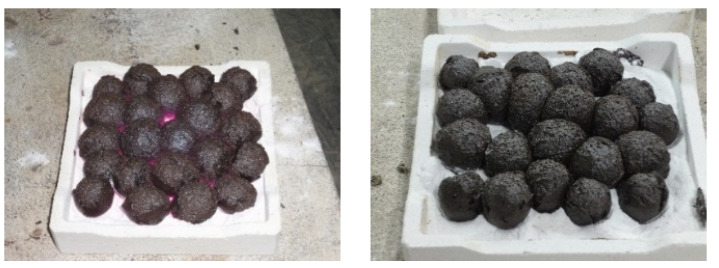
The overfiring phenomenon in the pellets.

Generally, the high-temperature properties of the pellets created with each formula were consistent with those composed of general materials and were sintered from the outside to the inside as the sintering temperature increased. In other words, a higher sintering temperature increases the melting of the material, thereby increasing the black core portion inside the aggregate (as shown in Figure 6 and Figure 7), which is consistent with the findings of Han et al. [42]. The overfiring phenomenon occurred, which means that the material softened and deformed, causing the pellets to stick to each other, as shown in Figure 8. 

The sintering temperature range of each formula was from the sintering temperature at which the pellet shrank the least in the temperature conditions at which the pellet underwent softening deformation, which was about 1075–1225 °C. As shown in Figure 6, the sintering temperature point of the W30 sample was about 1075 °C, and the softening temperature point was about 1225 °C. In addition, as the content of industrial sludge increased, its softening temperature decreased, and the formation of expansive interconnected giant pores and overfiring occurred easily. This is because the (SiO_2_ + Al_2_O_3_)/fluxing ratio of the W30 formula was higher than that of the W50 formula. Usually, a high (SiO_2_ + Al_2_O_3_)/fluxing ratio will result in a viscous liquid phase. This is similar to the findings of Chen et al. [14]. Based on the above observations and analysis, the formulations for sintering LWA as well as its sintering temperature range and softening temperature are summarized in Table 6. It should be noted that the W50 formula had a very narrow sintering temperature range. 

#### 3.1.2. The Second-Stage Trial Firing

According to the results of the first-stage trial firing, the sintering temperature range that was suitable for each formulation could be determined. Therefore, expansion phenomena observations were made for the formulations listed in Table 5. In addition, through the adjustment of the soaking time, the expansion properties and trends of each formula could be generally identified. When designing the sintering temperature, the softening temperature was used as the set value. In the preheating phase, the preheating temperature was set to 500 °C, and the soaking time was varied (5, 7, or 10 min). During the sintering phase, under the aforementioned conditions, the sintering temperature was fixed (depending on the softening temperature of each formulation), and the soaking time was set to 10 min.

**Figure 9:**
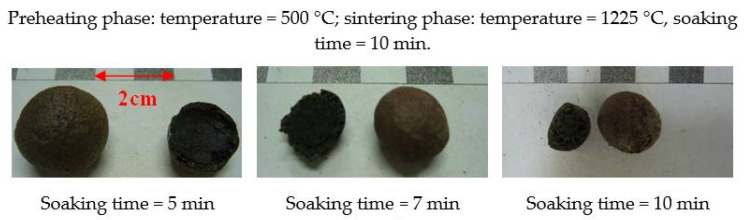
Appearances and profiles of the sintered LWAs during the second stage (the W30 formula).

**Figure 10:**
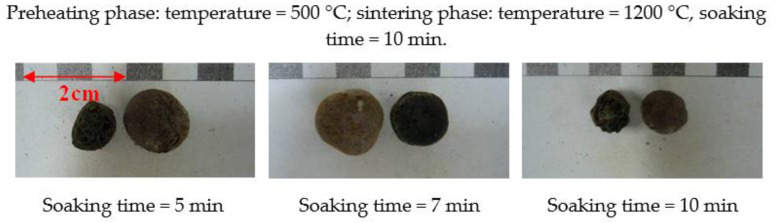
Appearances and profiles of the sintered LWAs during the second stage (the W50 formula).

The test results are shown in Table 7 and Figure 9 and Figure 10. The soaking time in the preheating phase had a great influence on the test results. A longer soaking time causes more organic matter in the raw material to decompose, forming a gas and escaping, resulting in the poor expansion of the sintered LWAs. In other words, a longer soaking time will lead to an increase in the particle density of the sintered LWAs. It can be seen from these results that regardless of how much sludge was added, its volume expansion (including visible pores) decreased as the soaking time increased, whereas the particle density increased as the soaking time increased. Furthermore, the bloating index was smaller as the amount of added sludge increased. The results show that the W30 formula with low sludge content had a better expanding capacity. This is similar to the findings of Chen et al. [24]. According to these results, the properties of the aggregates can be adjusted by increasing or decreasing the soaking time or the amount of sludge during firing applications in the future.

#### 3.1.3. The Third-Stage Trial Firing

After the first and second stages of trial firing, the characteristics of industrial sludge mixed with reservoir sediments for sintering LWA could be approximated. In order to further determine the physical properties and applicability of the obtained aggregates, on the basis of the results of the second-stage trial firing, more firing was carried out on the sample formulation and firing parameters with a larger bloating index. In addition, the physical properties of the obtained aggregates were also measured. At this stage, a total of three experimental formulations were developed, and the sludge proportions (accounting for the total weight of raw materials) were 10%, 30%, and 50%, as shown in Table 5. In the preheating phase, the preheating temperature was fixed at 500 °C; there were two different soaking times (i.e., 5 and 15 min). During the sintering phase, the sintering temperature was between 1200 and 1250 °C; there were three different soaking times (i.e., 5, 10, and 15 min).

**Figure 11:**
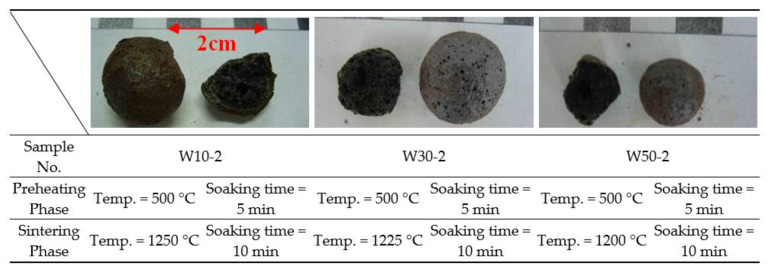
Appearances and profiles of the sintered LWAs during the third stage.

Under the conditions of the above firing parameters, their influence on the properties of the LWA obtained could be explored. In addition, the setting of the sintering temperature of each sample was determined on the basis of the softening temperature measured before the test, as shown in Table 8. The appearance and internal pore distribution of some of the aggregates obtained from the trial firing are shown in Figure 11. Overall, the loss on ignition, water absorption, particle density, and bloating index of the sintered LWAs are in good agreement with the literature results for similar materials [43,44,45]. Taking particle density as an example, the results of this study ranged from 0.41 to 1.63 g/cm^3^, while those of Souza et al. ranged from 0.63 to 2.01 g/cm^3^. The analysis of various aggregate properties is described in the following subsections.

**Figure 12:**
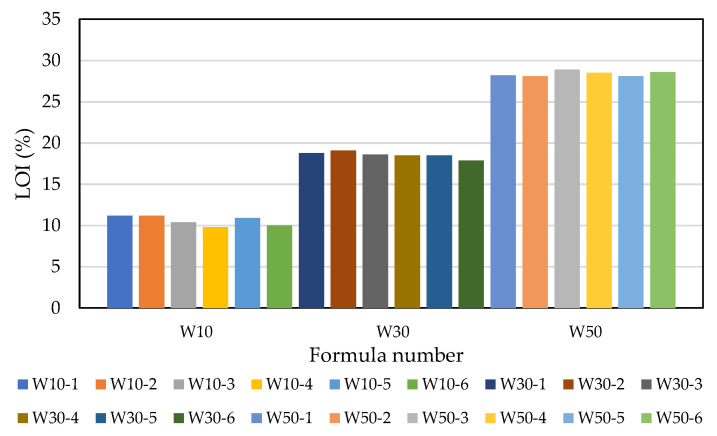
Loss on ignition analysis of each sample.

**Figure 13:**
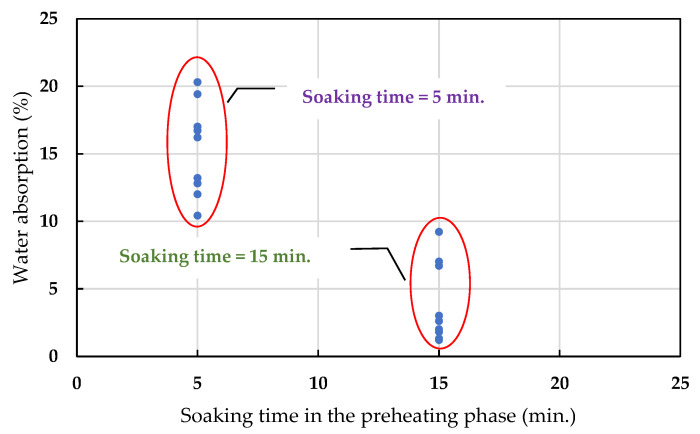
Influence of the soaking time during the preheating phase on the water absorption of the aggregate.

**Figure 14:**
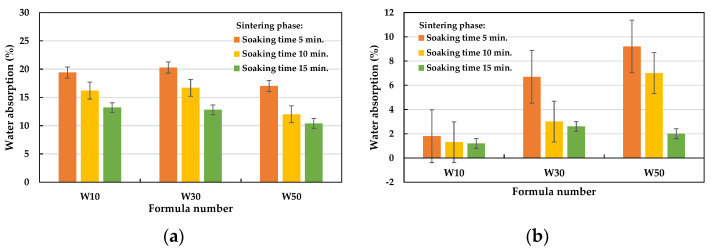
Influence of the soaking time during the sintering phase on the water absorption of the aggregate: (**a**) soaking time = 5 min during the preheating phase and (**b**) soaking time = 15 min during the preheating phase.

The weight reduction observed in the green pellets after firing is called the loss on ignition, which can be used as a basis for controlling the amount of LWA firing. It can be seen from Figure 12 that the loss on ignition increased as the amount of added sludge increased (averages of 10.6% for the W10 formula, 18.6% for the W30 formula, and 28.4% for the W50 formula). Table 8 shows the 24 h water absorption of the LWA prepared with samples of each formula under different firing conditions. Based on the amount of sludge added, the water absorption rates of the aggregates in the W10, W30, and W50 formulations ranged from 1.2 to 19.4%, 2.6 to 20.3%, and 2.0 to 17.0%, respectively. In addition, the soaking time used in the preheating phase has a great influence on the water absorption of the aggregate. Under the same test conditions, regardless of the amount of sludge added, the longer the soaking time in the preheating phase, the lower the water absorption rate of the LWA obtained; as shown in Figure 13, the water absorption rate of the aggregate was less than 10% when the soaking time in the preheating phase was 15 min. On the other hand, the longer the soaking time in the sintering phase, the lower the water absorption of the resulting aggregate, as shown in Figure 14. Regardless of whether the soaking time was 5 min or 15 min during the preheating phase, the water absorption of all of the samples decreased with the increase in the soaking time in the sintering phase. Furthermore, it can be seen from Figure 14b that under the condition of a 15 min soaking time during the preheating phase, when the added sludge content was higher, the water absorption capacity of the fired LWA was higher (i.e., W10 < W30 < W50).

**Figure 15:**
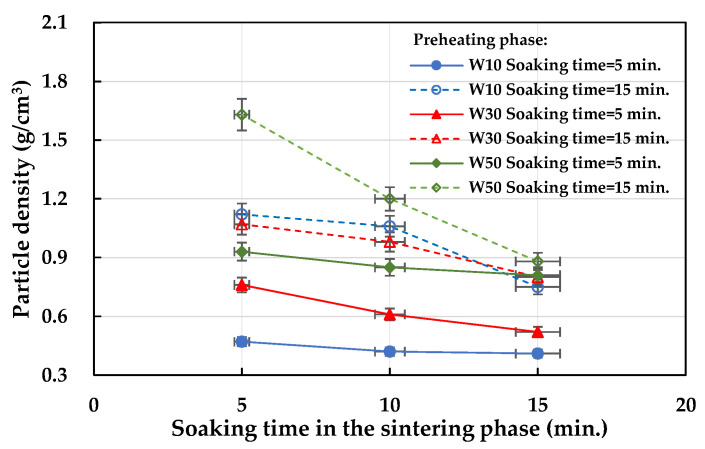
Influence of the soaking time during the preheating phase on the particle density of the aggregate.

**Figure 16:**
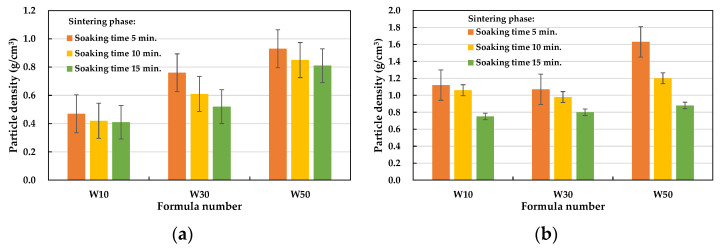
Influence of the soaking time during the sintering phase on the particle density of the aggregate: (**a**) soaking time = 5 min in the preheating phase and (**b**) soaking time = 15 min in the preheating phase.

The particle density test results of the fired LWA are also listed in Table 8. With the amount of the sludge added as a distinction, the particle density of the LWA in the W10, W30, and W50 formulations ranged from 0.47 to 1.12, 0.52 to 1.07, and 0.81 to 1.63 g/cm^3^, respectively. These results show that the designed experimental formulations are suitable for LWA firing. In terms of the effect of the soaking time during the preheating phase on the particle density of the LWA, under the same formula and firing conditions, the longer the soaking time during the preheating phase (i.e., 15 min), the greater the particle density (see Figure 15). In addition, the longer the soaking time during the sintering phase, the lower the density of the obtained LWA, as shown in Figure 16. Regardless of whether the soaking time was 5 or 15 min during the preheating phase, the particle densities of all of the samples decreased as the soaking time during the sintering phase increased. Furthermore, it can be seen from Figure 16a that under the conditions of a 5 min soaking time during the preheating phase, the lower the amount of sludge added, the lower the particle density (i.e., W10 < W30 < W50).

**Figure 17:**
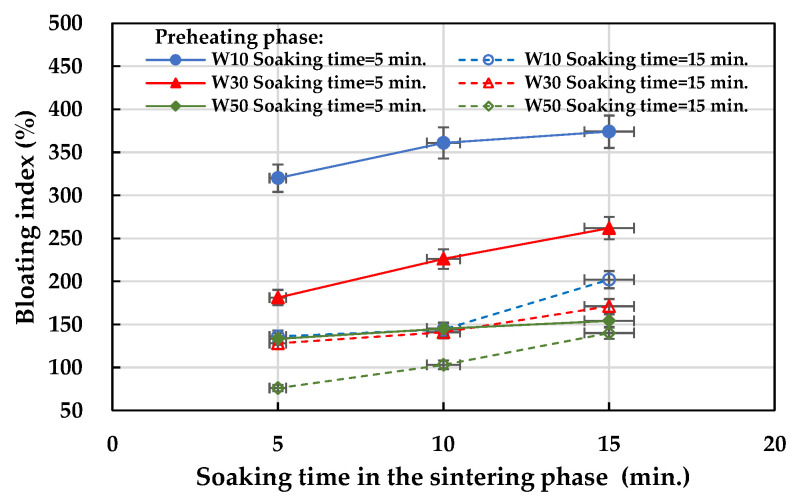
Influence of the soaking time during the preheating phase on the bloating index of the aggregate.

**Figure 18:**
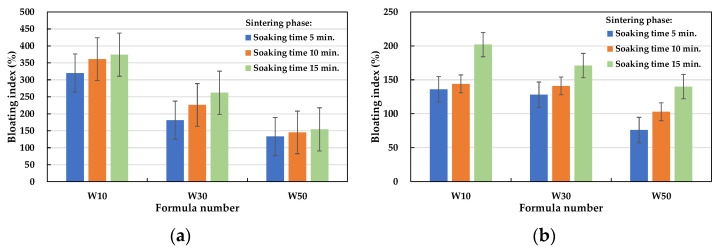
Influence of the soaking time during the sintering phase on the bloating index of the aggregate: (**a**) soaking time = 5 min in the preheating phase and (**b**) soaking time = 15 min in the preheating phase.

The test results of the bloating index of the fired LWAs are listed in Table 8. Based on the amount of the sludge added, the bloating index of the aggregates fired with the W10, W30, and W50 formulations ranged from 136 to 374%, 128 to 262%, and 76 to 154%, respectively. These results show that most of the designed formulations were suitable for firing expanded LWA. In terms of the effect of the soaking time during the preheating phase on the bloating index of the aggregate, under the same formula and firing conditions, the longer the soaking time during the preheating phase (i.e., 15 min), the smaller the bloating index (see Figure 17). In addition, the longer the soaking time during the sintering phase, the larger the bloating index of the obtained LWA, as shown in Figure 18. Regardless of whether the soaking time was 5 or 15 min during the preheating phase, the bloating index of all of the samples increased as the soaking time during the sintering phase increased. Furthermore, it can be seen from Figure 18a that under the conditions of a 5 min soaking time during the preheating phase, the smaller the amount of sludge added, the larger the bloating index (i.e., W10 > W30 > W50). In sum, regardless of how much industrial sludge was added, the water absorption rate and bloating index of the fired LWA decreased as the soaking time increased during the preheating phase. For the same formula, the increase in the soaking time during the sintering phase resulted in a lower particle density and water absorption. The particle densities of the aggregates fired with the addition of 10%, 30%, and 50% ranged from 0.41 to 1.12, 0.52 to 1.07, and 0.81 to 1.63 g/cm^3^, respectively. This shows that the above formulation is suitable for firing LWA for the purposes of thermal insulation. Furthermore, the larger the amount of added sludge, the larger the particle density and the loss on ignition, and the smaller the bloating index. The water absorption rate was distributed between approximately 2% and 20%.

### 3.2. Feasibility of Large-Scale Production and Application in Concrete

#### 3.2.1. Mass-Produced Formulas

The laboratory-scale firing results show that the sludge from wastewater treatment plants contains a large number of oxides that are common in nature, such as silicon, aluminum, iron, and calcium. In other words, its chemical composition is similar to that of ceramic materials, and it has the potential to make lightweight aggregates. Therefore, in this study, the sludge from the Sinying Industrial Park Wastewater Treatment Plant was further incorporated into clay, and existing commercial rotary kiln process equipment was used for sintering to mass-produce LWAs for construction projects. During the large-scale trial firing, four experimental formulations were developed, and the proportions of sludge added were 0%, 30%, 40%, and 50%, as shown in Table 9. Among them, the W0 formula was used as the control group, and the other three formulas were used as the experimental group.

#### 3.2.2. Mass Production Process

**Figure 19:**
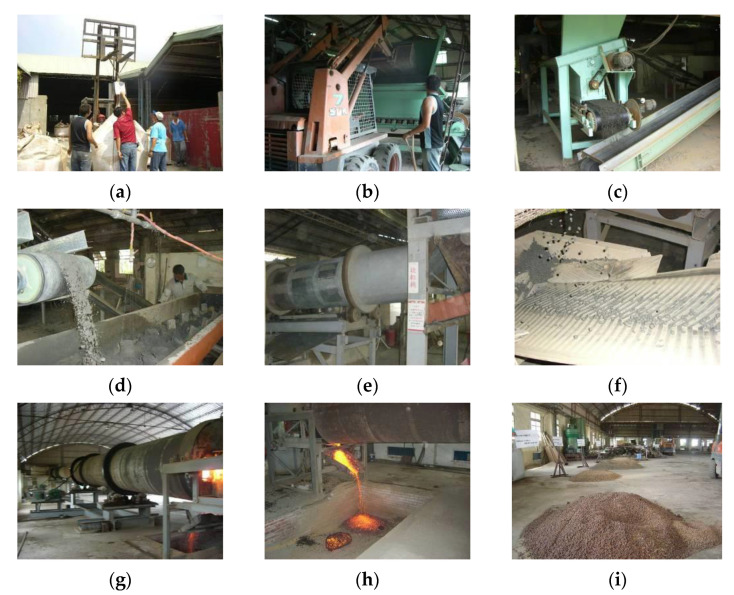
LWA mass production operation process: (**a**) weighing, (**b**) shoveling, (**c**) crushing, (**d**) mixing, (**e**) graining, (**f**) green pellet (**g**) sintering, (**h**) unloading, and (**i**) cooling.

The operation flow of firing with the rotary kiln is shown in Figure 19. First, according to Table 9, the amounts of sludge and clay in the mixture were individually weighed, as shown in Figure 19a. Next, the raw materials were sent to the feeder by a scraper, and the attached crusher was used for crushing and mixing, as shown in Figure 19b,c, respectively. Afterwards, the mixed materials were sent to the kneader by a conveyor belt, and an appropriate amount of water was added for uniform mixing and kneading, as shown in Figure 19d. Then, the above-mentioned prepared materials were sent to a granulator to be extruded into cylindrical green pellets of the same size (8–10 mm in diameter), as shown in Figure 19e,f. The firing kiln used in this study (as shown in Figure 19g) can be divided into a preheating section and a firing section. The preheating section was mainly to dry the pellets; the temperature was about 200–800 °C, and the soaking time of the sample was about 20–25 min. The temperature of the sintering section was 1000–1250 °C, mainly to provide a higher temperature for swelling the pellets. The sample soaking time was 20–30 min, and the actual soaking time could be adjusted according to the characteristics of the product. The sintered LWAs were removed from the kiln through the discharge port, as shown in Figure 19h. They were then sent to the cooling area by the scraper, and the heat was gradually released by the natural cooling method, as shown in Figure 19i. The firing parameters determined after the mass production test are shown in Table 10.

#### 3.2.3. Test and Analysis of Physical Properties of the Sintered Aggregates

Table 11 summarizes the quality requirements of the Chinese National Standards (CNS) for LWA products. Among them, the dry loose bulk density of LWAs must be less than 880 kg/m^3^ in order to comply with the CNS 3691 [46] “Lightweight aggregates for structural concrete” and CNS 14,826 [47] “Lightweight aggregates for insulating concrete”. The upper limit of water absorption is set at 15%. In addition, according to CNS 14779 [41] “Method of test for the particle cylindrical crushing strength of lightweight coarse aggregates”, the crushing strength of lightweight aggregates must be measured; the difference between the maximum value and the minimum value must be less than 15% of the average value to ensure the quality of aggregate strength.

**Figure 20:**
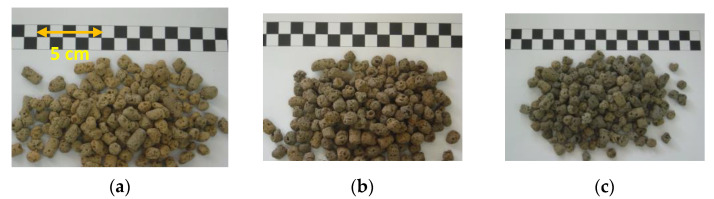
Appearances of the sintered LWAs during large-scale trial firing: (**a**) the W30 formula, (**b**) the W40 formula, and (**c**) the W50 formula.

**Figure 21:**
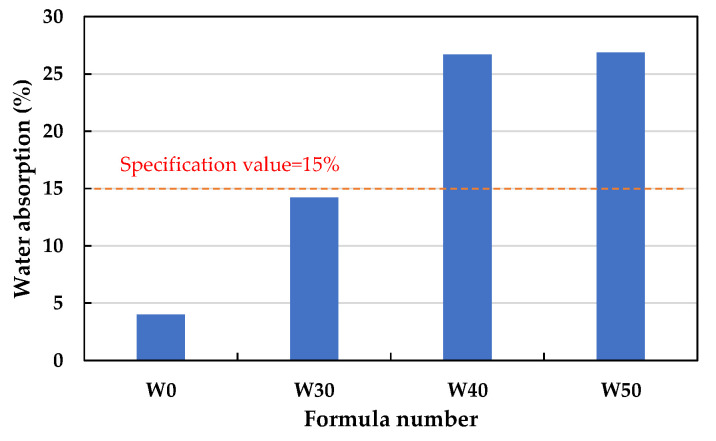
Water absorption of the sintered LWAs.

Table 12 summarizes the test results for the water absorption, dry loose bulk density, and crushing strength of the finished LWAs fired with the four formulations. It can be seen from Table 12 that the water absorption, dry loose bulk density, and crushing strength of the fired LWAs were 14.2–26.9%, 634–753 kg/m^3^, and 1.29–2.90 MPa, respectively. In addition, the appearances of the manufactured LWAs in the experimental group are shown in Figure 20. As can be seen from the figure, the LWAs have visible pores. As shown in Figure 21, the water absorption of the LWAs increased as the amount of added sludge increased. Among the formulations, the water absorption of the LWAs produced by incorporating 30% sludge was less than the upper limit of the product quality standard (15%). The water absorption of the LWAs produced by incorporating 40% and 50% sludge was significantly higher than the upper limit of the product quality standard.

**Figure 22:**
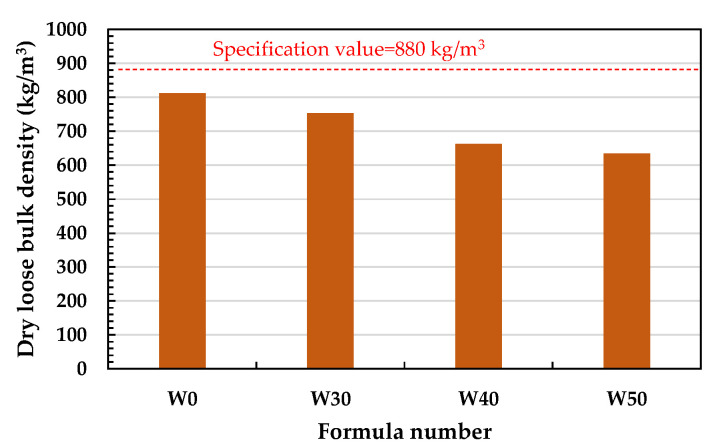
Dry loose bulk density of the sintered LWAs.

**Figure 23:**
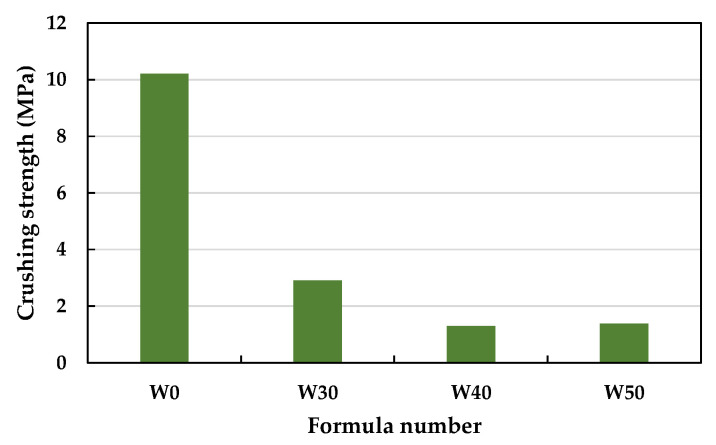
Crushing strength of the sintered LWAs.

As shown in Figure 22, the dry loose bulk density of the fired LWAs gradually decreased as the amount of added sludge increased; that is, it increased from 812 kg/m^3^ for the W0 formula to 634 kg/m^3^ for the W50 formula. Figure 22 shows that the dry bulk densities of the LWAs fired by the three different sludge blend percentages in the experimental group were all lower than 880 kg/m^3^, meeting the requirements of CNS 3691 and CNS 14826. In addition, Figure 23 shows that the crushing strength of the fired LWAs also decreased as the amount of added sludge increased; that is, from 2.90 MPa in the W30 formulation to 1.29–1.38 MPa in the W40 and W50 formulations. As seen in Figure 23, the crushing strength of the LWAs fired by the three different sludge blend percentages in the experimental group was significantly lower than that of W0. Among the samples, the crushing strength of the LWAs produced by mixing 30% sludge could be maintained at about 2.90 MPa, and the difference between the maximum value and the minimum value was less than 15% of the average value, which meets the established product quality standards. 

The test results of the heavy metal toxicity characteristic leaching procedure (TCLP) of the sludge and LWAs are shown in Table 13. It can be seen from Table 13 that the results of the heavy metal TCLP dissolution test for the LWAs produced by blending 30–50% of the sludge were all lower than the standard value required by the Taiwan Environmental Protection Agency for general industrial waste. This result shows that a high-temperature (1200 °C) sintering treatment could effectively seal and stabilize harmful heavy metal substances, which is consistent with the findings of Li et al. [48]. As reported in Table 12, the water absorption, dry loose bulk density, and crushing strength were 14.2%, 753 kg/m^3^, and 2.90 MPa, respectively, for the LWAs produced by mixing 30% sludge. In addition, the difference between the maximum value and the minimum value of the crushing strength was less than 15% of the average value. These results are in line with the established product quality standards. Considering that the products will be mainly used in the production of concrete products for construction projects in the future, the upper limit of the mixing ratio should be 30% sludge when reused for future applications. For the sintered LWAs comprising 40% and 50% sludge, the water absorption was greater than the preset product standard value, which is not appropriate for final remanufactured products (such as ready-mixed concrete production). However, it has high water absorption (>26%) and is light weight (unit weight of dry pine < 670 kg/m^3^), and it can be applied to geotechnical engineering applications, such as in site improvement projects or sustainable urban drainage systems.

#### 3.2.4. Properties of Concrete Made from the Sintered LWAs

**Figure 24:**
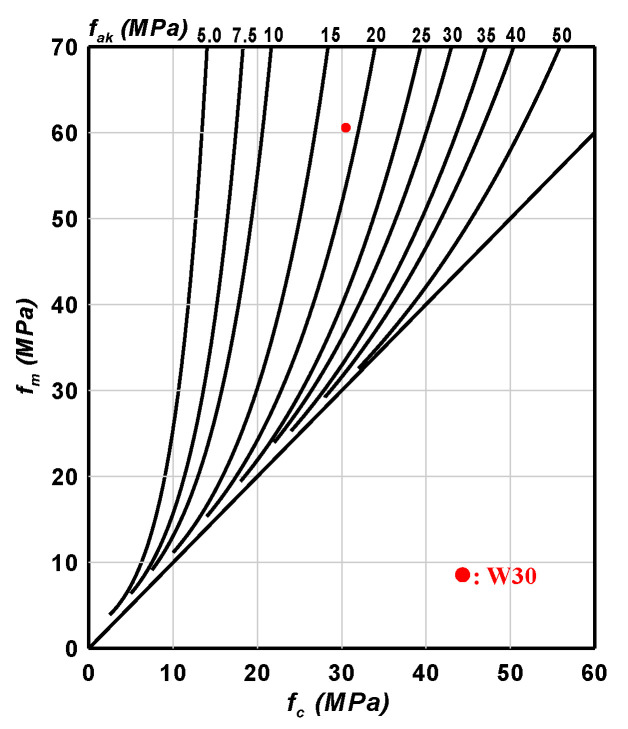
Test result of the strength grade of the sintered LWAs.

In order to determine the achievable strength of concrete made from the sintered LWAs, an analysis of the attainable strength of concrete made with lightweight sludge aggregates was carried out. The analysis was mainly based on the Chinese GB/T 17431.2-1998 [49] “Lightweight Aggregate Strength Grade Test Method” to evaluate the compressive strength properties and feasibility of LWA applied to concrete. The concrete mix design used in this test is shown in Table 14, and the strength grade test results are shown in Table 15 and Figure 24. Based on the measured test results for mortar strength (*f_m_*) and concrete strength (*f_c_*), a solid red circle can be drawn between the reasonable concrete strength (*f_ak_*) lines at 15 and 20 MPa, as shown in Figure 24. Therefore, the strength grade of the sintered LWA was 20 MPa. From this point of view, the sintered LWA produced under the test conditions can be used as an aggregate in lightweight concrete, which would have a reasonable strength of greater than 20 MPa, meeting the requirements for concrete strength for general building structures.

## 4. Conclusions

In this study, the sustainable use of sludge from industrial park wastewater treatment plants in manufacturing LWAs was explored. The main goal of the study was to conduct laboratory-scale and large-scale tests to determine optimum sintering temperature, sintering time, and raw material formulation of sintered aggregates by incorporating industrial wastewater sludge with reservoir sediments or clay. The results of the study showed that the water absorption of the sintered LWAs increased as the percentage of added sludge increased. However, only the water absorption of the LWAs produced by mixing 30% sludge was less than 15%, whereas the water absorption of the LWAs produced by mixing 40% and 50% sludge was markedly higher. In addition, the dry loose bulk density of the sintered LWAs gradually decreased as the percentage of additional sludge increased. The dry loose bulk densities of the sintered LWAs with the three different sludge addition percentages were all less than 880 kg/m^3^, which is in compliance with Chinese national standards CNS 3691 and CNS 14826. If considering the use of LWAs in the production of concrete products in construction projects, the percentage of sludge added should be limited to 30%. The sintered LWAs with 40% and 50% sludge have the characteristics of high water absorption (>26%) and light weight (dry loose unit weight < 670 kg/m^3^), which can be applied to geotechnical engineering applications such as site improvement projects or sustainable urban drainage systems.

On the other hand, the results of the heavy metal TCLP dissolution test for the LWAs produced by blending 30–50% of the sludge were all lower than the standard value required by the Taiwan Environmental Protection Agency for general industrial waste. This result shows that a high-temperature (1200 °C) sintering treatment could effectively seal and stabilize harmful heavy metal substances. Moreover, the strength grade of the sintered LWA was 20 MPa. From this point of view, the LWA that was sintered under the test conditions could be used as an aggregate in lightweight concrete, which would have a reasonable strength of greater than 20 MPa, which meets the requirements for general building structures constructed with concrete strength. 

The research results showed that the organic sludge from Sinying Industrial Park Wastewater Treatment Plant had the potential to be used as an auxiliary raw material for LWA. Compared to the early marine disposal and burial treatment of sludge, this study greatly improves the high added value of sludge materialization applications. Lightweight aggregate is a building material that is lightweight, has good fire resistance, and good heat insulation, but lightweight aggregate fired at a high temperature has a certain impact on the environment. How to adjust the composition of lightweight aggregate raw materials to reduce energy consumption is the focus of future research? In addition, the process technology of lightweight aggregates must also be improved, such as through a switch to clean energy, in response to the energy conservation and carbon reduction policies advocated by countries around the world.

## Figures and Tables

**Table 1:** Classification of lightweight aggregates.

Aggregate Type		Particle Diameter (mm)	Bulk Density (kg/m^3^)
Coarse LWA		≥5	<1000
Fine LWA	Natural aggregate	<5	<1000
	Artificial aggregate	<5	<1200

**Table 2:** Types and basic physical properties of lightweight aggregates.

Source	Type	Physical Properties			
		Bulk Density (g/cm^3^)	Particle Density (g/cm^3^)	Porosity (%)	Water Absorption (%)
Natural	Pumice	0.34–0.63	0.35–1.15	85	up to 50
	Lava	0.75–1.4	1.8–2.8	40	10
Artificial	Perlite	0.04–0.15	0.1–0.3	95	≒0
	Vermiculite	0.06–0.17	0.1–0.35	90	≒0
	Expanded clay	0.3–0.9	0.6–1.8	75	8–20
	Expanded shale	0.45–0.9	0.8–1.8	70	5–10
	Expanded slag	0.5–0.85	1.0–2.0	46–60	20–35
	Organic foam balls	0.02	0.04	99	≒0

**Table 3:** Physical properties of the materials used.

Physical Property	Reservoir Sediments	Sludge
D_50_ (mm)	0.004	0.11
Plastic index (%)	12	4
Specific gravity	2.75	1.74

**Table 4:** General characteristics of the materials used.

Item	Reservoir Sediments	Sludge
SiO_2_ (%)	62.1	18.1
Al_2_O_3_ (%)	6.27	19.2
CaO (%)	1.16	4.03
MgO (%)	1.73	0.92
K_2_O (%)	0.0066	0.0686
Na_2_O (%)	3.42	0.13
Fe_2_O_3_ (%)	6.43	6.15
Moisture (%)	7.91	21.09
Ash (%)	87.66	33.96
Combustible (%)	4.43	44.95
Organic matter	2.6	41.9
SO_4_ (%)	0.05	0.28
Cl^−^ (%)	0.04	0.07

**Table 5:** Experimental LWA formula.

Formula Number	Proportion of Mixture (% wt.)		Granulation Feasibility
	Sludge	Reservoir Sediments	
W10	10	90	Feasible
W30	30	70	Feasible
W50	50	50	Feasible
W70	70	30	Fail

**Table 6:** Sintering temperature range and softening temperature of each formula.

Formula Number	Sintering Temperature Range	Softening Temperature
W30	1075–1225 °C	1225 °C
W50	1075–1200 °C	1200 °C

**Table 7:** Physical properties of aggregates in each formulation at the second stage.

Formula Number	Preheating Phase		Sintering Phase		Particle Density (g/cm^3^)	Bloating Index (%)
	Temperature (°C)	Soaking Time (min)	Temperature (°C)	Soaking Time (min)		
W30	500	5	1225	10	0.61	225.75
		7			0.64	213.84
		10			0.98	140.64
W50		5	1200		0.82	152.04
		7			0.85	145.21
		10			1.08	128.08

**Table 8:** Physical properties of aggregates in each formulation during the third stage.

Sample No.	Preheating Phase		Sintering Phase		LOI (%)	Water Absorption (%)	Particle Density (g/cm^3^)	Bloating Index (%)
	Temp. (°C)	Soaking Time (min)	Temp. (°C)	Soaking Time (min)				
W10-1	500	5	1250	5	11.2	19.4	0.47	320
W10-2				10	11.2	16.2	0.42	361
W10-3				15	10.4	13.2	0.41	374
W10-4		15		5	9.8	1.8	1.12	136
W10-5				10	10.9	1.3	1.06	144
W10-6				15	10.0	1.2	0.75	202
W30-1	500	5	1225	5	18.8	20.3	0.76	181
W30-2				10	19.1	16.7	0.61	226
W30-3				15	18.6	12.8	0.52	262
W30-4		15		5	18.5	6.7	1.07	128
W30-5				10	18.5	3.0	0.98	141
W30-6				15	17.9	2.6	0.80	171
W50-1	500	5	1200	5	28.2	17.0	0.93	133
W50-2				10	28.1	12.0	0.85	145
W50-3				15	28.9	10.4	0.81	154
W50-4		15		5	28.5	9.2	1.63	76
W50-5				10	28.1	7.0	1.20	103
W50-6				15	28.6	2.0	0.88	140

Note: LOI = Loss of ignition.

**Table 9:** The amount of sludge and clay required for each formulation.

Formula Number	Proportion of Mixture (% wt.)		Sludge (Dry) (kg)	Clay (Dry) (kg)	Sludge (Wet) (kg)	Sediments (Wet) (kg)	Mixing Amount (kg)
	Sludge	Sediments					
W0	0	100	0	3518	0	3820	3820
W30	30	70	1005	2345	1273	2547	3820
W40	40	60	1318	1979	1671	2149	3820
W50	50	50	1623	1623	2056	1764	3820
Total			3946	9465	5000	10,280	15,280

Note: The moisture content of sludge on a wet basis was 21.09%; the moisture content of clay on a wet basis was about 7.91%.

**Table 10:** Firing parameters (calibration).

Rotary Kiln Operation Record					
Time When Entering the Kiln	Exhaust Volume	Preheating Section	Sintering Section		The Time Out of the Kiln
	Number of Gates	Rotating Speed (RPM)	Rotating Speed (RPM)	Temperature (°C)	
08:05	4	3.5	1	1010	08:55

**Table 11:** Quality requirements for LWA products.

Property	Quality Requirements	Code
Dry loose bulk density	<880 kg/m^3^	CNS 3691 and CNS 14826
Water absorption	<15%	-
Crushing strength	The difference between the maximum value and the minimum value is less than 15% of the average value.	CNS 14779

**Table 12:** Properties of the sintered LWAs.

Formula Number	Property		
	Water Absorption (%)	Dry Loose Bulk Density (kg/m^3^)	Crushing Strength (MPa)
W0	4.0 ± 0.2	812 ± 6	10.21 ± 0.21
W30	14.2 ± 0.1	753 ± 8	2.90 ± 0.14
W40	26.7 ± 1.2	662 ± 4	1.29 ± 0.01
W50	26.9 ± 0.3	634 ± 4	1.38 ± 0.01

**Table 13:** TCLP results of the raw materials and LWA.

Formula Number	Heavy Metals								
	Cu (mg/L)	Cd (mg/L)	Cr (mg/L)	Pb (mg/L)	As (mg/L)	Hg (mg/L)	Se (mg/L)	Cr^6+^ (mg/L)	Ba (mg/L)
Sludge	0.109	ND	<0.020	ND	ND	ND	<0.100	<0.10	0.328
Clay	<0.020	ND	<0.020	<0.040	ND	ND	<0.100	<1.00	0.725
W30	<0.020	ND	<0.020	<0.040	ND	ND	<0.100	<0.10	0.321
W40	0.139	ND	<0.020	0.114	0.272	ND	<0.100	<1.00	0.191
W50	0.032	ND	<0.020	<0.040	0.348	ND	<0.100	<1.00	0.267
Specification value *	15	1	5	5	5	0.2	1	2.5	100

Note: ND = not detected; * the standard for hazardous industrial wastes, EPA, Taiwan.

**Table 14:** Mix proportion of concrete using the sintered LWAs.

Water/Cement (W/C)	Water (kg/m^3^)	Cement (kg/m^3^)	Fine Aggregate (kg/m^3^)	LWA (kg/m^3^)	Unit Weight (kg/m^3^)
0.4	202	504	504	777	1987

Note: According to the specifications, the calculation value of the proportion of freshly mixed cement mortar is set with a unit weight of 2200 kg/m^3^.

**Table 15:** Results of the strength grade of the sintered LWA produced in the commercial rotary kiln.

Sample Group	28-Day Compressive Strength (MPa)	Strength Grade (MPa)	Specification Value (MPa)
W30	31	20	20
Cement mortar	61	—	—

Note: The specification value is the strength grade that should be achieved according to its aggregate density grade.

## Data Availability

The data presented in this study are available upon request from the corresponding author.
